# Small samples and increased variability – discussing the need for restricted types of randomization in exercise interventions in old age

**DOI:** 10.1186/s11556-019-0224-3

**Published:** 2019-10-27

**Authors:** Yael Netz, Ronnie Lidor, Gal Ziv

**Affiliations:** The Academic College at Wingate, Wingate Institute, Netanya, Israel

**Keywords:** Unequal groups, Biased results, Stratified randomization, Small study groups, Probability of baseline differences

## Abstract

**Background:**

Randomization provides an equal chance for participants to be allocated to intervention groups, in order to create an equal distribution of all variables at baseline in all groups. However, this is not guaranteed, particularly if the groups are too small, or if the researched groups consist of older adults. The aims of this commentary are to discuss the increased variability in old age which intensifies the risk of baseline inequalities, to elaborate on the need to estimate potential baseline group differences in small samples of older participants in exercise intervention, to discuss alternative procedures for creating equal groups at baseline and to provide specific guidelines for selecting the design of small studies.

**Main body:**

Small groups with increased inter-individual differences may lead to reduced power, thus differences that truly exist may not be detected, or false group differences may appear in the outcome following the treatment. Studies that focused exclusively on older adults have found increased variability in advanced age. Therefore, baseline group differences are more common in older adults as compared to younger persons, and may lead to misinterpretation of the intervention′s results. Imbalances can be reduced by covariate-adaptive randomization procedures, such as stratified permuted-block randomization or minimization. Specific guidelines are provided for selecting a randomization procedure by assessing the probability of unequal groups at baseline in typical, widely used functional tests in old age. A calculation of the required number of participants for creating equal groups for these functional tests is provided, and can be used when increasing the number of participants is possible. R-scripts specifically created for assessing the probability of unequal groups, or for determining the sample size assuring equal groups, are recommended.

**Conclusions:**

In exercise interventions assessing older adults, it is recommended to have a sample large enough for creating equal groups. If this is not possible, as is the case quite often in intervention studies in old age, it is recommended to assess the probability of inequality in the study groups and to apply an alternative randomization.

## Background

### Randomization does not imply equivalence in small samples

The allocation of participants to experimental conditions via a random procedure is considered to be a fundamental statistical technique in biomedical research. In fact, statistical experiments, including experiments in exercise sciences [1], are defined by the use of randomization [2]. This means that each participant has an equal chance to be assigned to any of the study groups, and this is assured by such means as a coin toss or a table of random numbers.

The value of a control group for determining that the intervention is the only explanation for the change in the experimental group depends on its similarity to the experimental group [1]. Thus, in order to protect against bias, the objective of randomization is to have two groups that are as equal as possible [3]. This helps to ensure that the treatment groups being compared are similar in both measured and unmeasured participant characteristics [4]. It is therefore not surprising that the randomized experiment is often referred to as the gold standard of research – specifically clinical research, including exercise training research [1]. Indeed, many applied fields, for example evidence-based medicine, draw a basic distinction between randomized and non-randomized evidence [5].

However, an equal chance to be allocated to each of the experimental groups does not ensure group equality [4, 6]. That is, it does not guarantee balance in covariate distributions across treatment groups [6]. Imbalanced groups may occur particularly when there are relatively few participants (e.g. 15 to 20 participants per group) enrolled in a trial [4], with one paper even claiming that less than 100 participants in a group can be a problem for achieving equality [7].

In a previous article, we examined the use of stratified permuted-block randomization as an alternative procedure for simple randomization in the case of unequal groups at baseline in motor learning research [8]. The aims of the present commentary are: 1) to discuss the increased variability in old age, which intensifies the risk of baseline inequalities; 2) to elaborate on the need to estimate potential baseline group differences in small samples of older participants in exercise interventions; 3) to discuss alternative procedures for creating equal groups at baseline in these interventions; and 4) to provide specific guidelines for the design of small samples in these interventions.

## Main text

### Increased variability in old age

In exercise sciences, age is a moderating variable in almost all areas of research, primarily in relation to physical variables but also to psychological, social and behavioral variables, which are often studied in relation to exercise or fitness. One study examining the relationship between chronological and biological age showed that the variability in physical deterioration starts as early as age 38 [9]. Advancing age is typified by increasing variability in both physical [e.g. 10] and mental [e.g. 11] fitness between individuals. Therefore, in clinical interventions in exercise science age should be clearly defined, and if the range of the participants’ ages is large, then the participants should be divided into age groups and the intervention will be assessed per age group.

Even studies focusing exclusively on older adults have found that inter-individual variability shows a quantitative increase in advanced age in both physical and behavioral aspects. For example, a study on gait measures in people aged 60–86 reported a relationship between age and gait variability after adjustment for height, weight and chronic disease. Older age was associated with greater variability in all gait measures that are commonly associated with falls [10]. Another study assessing postural stability in older adults reported an age variability within this age group on static and dynamic balance, concluding that balance performance is task-specific in older adults. Thus, it was recommended that various dynamic and static balance tests be used for assessing postural balance ability in old age [11]. As postural stability is known to be specifically sensitive to age, normative data provided for a unipedal balance test (one-leg stance), for example, were divided into subgroups of 5 years each (65–69, 70–74, 75–79, etc.) [12].

Age variability in older adults was also indicated in the relationship between postural control and specific aspects of cognition, such as attention control [13]. Increased variability in old age has also been reported in response to aerobic exercise interventions [14], executive functioning [15] and reaction time [16]. Specifically, increased variability in reaction time was described in relation to aerobic fitness [17], falls and gait [18], and even to mortality in old age [19]. It has been argued that both inter- and intra-variability in reaction time was greater in older as compared with younger adults [20].

### The need to examine potential baseline group differences in small samples of aging populations

Given the increased variability in old age, it may be possible that after a procedure of simple randomization, participants in controlled studies will have baseline differences in the outcome measures or in other relevant covariates. This is particularly important in small groups of participants (e.g. 15–30 participants in each group), in which the number of covariates that can be balanced in stratified randomization is largely limited [6]. That is, in addition to the increased variability in old age, a small number of participants may indicate less statistical power. A study with high statistical power has a greater probability of detecting a specified treatment effect at any level of statistical significance. Statistical power is inversely related to variance of the difference between two means [7]. A study comprised of small groups with increased inter-individual differences may have a reduced power, and thus fail to detect differences that actually exist.

Furthermore, a bias that may occur in small groups with unequal baseline scores may lead to false group differences, in favor of the experimental group, in the outcome following the treatment. This may happen when the randomization process allocates participants in a way that the active treatment group has a better chance to improve than participants in the control group [7]. Specifically, it may occur when the baseline scores of the experimental group are lower than those of the control group – thus giving the experimental group “more room” for improvement.

Examples from the literature are presented in Table 1. In these examples participants were randomly allocated to experimental and control groups. However, the scores were not equal at baseline, with the experimental group scoring lower than the control group. In two studies [21, 23], the post-test scores of the experimental group were equal to the pre-test scores of the control group. The baseline differences between the experimental and control groups may have indicated the possibility that the controls did not improve because they were performing at ceiling level in terms of the potential range of improvement, whereas the exercise group had some room for improvement [23].

**Table 1:** Examples of small groups with unequal baseline scores with experimental groups’ scores lower than the control group

Study	Population (Age)	Measure	Group (n)	Pre-scores	Post-scores
Rehfeld et al., 2017 [21]	Healthy (63–80)	Composite equilibrium score (balance) %	Experimental (14)	~ 85.8	~ 87.6
			Control (12)	~ 87.45	~ 86.55
Gomes et al., 2018 [22]	Frail and pre-frail (71–92)	MiniBest (balance) total score	Experimental (15)	14.5 (±6.22)	18.5 (±7.95)
			Control (15)	17.4 (±6.87)	17.3 (±5.68)
Gomes et al., 2018 [22]	Frail and pre-frail (71–92)	Functional gait assessment total score	Experimental (15)	15.7 (±4.59)	18.8 (±5.75)
			Control (15)	17.1 (±6.32)	16.9 (±5.82)
Netz et al., 2007 [23]	Healthy (50–64)	Cognitive flexibility (Alternate Uses score)	Experimental (40) – *two experimental groups of 20 each – combined*	14.62 (±4.86)	16.9 (±4.88)
			Control (18)	16.94 (± 6.25)	16.39 (± 5.1)

In a previous article [8] we provided a flowchart and R scripts for examining the probability of inequality between groups at baseline, and for selecting an effective randomization strategy. Based on these R scripts, we calculated the chances for baseline group differences in the Gomes et al. [22] and Netz et al. [23] studies (this was not possible in the Rehfeld et al. study [21], as no SDs were provided). Assuming a mean of 16, a SD of 5 and 15 participants in each experimental group, we found a 66% chance for a 5% baseline group difference, a 38% chance for a 10% difference, and an 8% chance for a 20% difference (see Table 1). These calculations suggest that it is quite possible that the groups may have not been equal at baseline.

Thus, although these studies reported that the treatment was beneficial, it is likely that had the participants in the experimental groups performed better at baseline, their performance following the exercise treatment would have shown less improvement. Hence, it is possible that the treatment only helped those who had poor scores at baseline. In other words, the interaction indicating greater improvement for the experimental than the control group may not indicate that the treatment was actually beneficial.

### Alternative procedures for creating equal groups at baseline

Imbalances between groups can be reduced in small sample-size studies by restricting the randomization procedure. Restricted randomization means that simple randomization is applied within defined groups of participants [4, 24]. Covariate-adaptive randomization is the most commonly used procedure for creating balance in relevant covariates across treatment groups [6, 24].

The two leading techniques of covariate–adaptive randomization are stratified (block) randomization and minimization [1, 6, 24]. Stratified randomization controls treatment imbalances within each covariate stratum [24]. It creates a separate randomization process, usually a permuted block design, for each specific stratum formed by a combination of the levels of the relevant covariates [6]. For example, in studies assessing the effect of a certain exercise intervention on cognitive functioning, fitness level and gender will be typical strata. The limitation of this technique is that if the sample size is small, the number of strata is very limited. Too many covariates (strata) means a small number of participants in each stratum or even empty strata. If only a few strata are allowed the imbalance could be reduced, but would still be present [7].

While stratified randomization is a predetermined technique, with participants being assigned to groups in advance, minimization enables an on-going process of group allocation. Minimization achieves balance in treatment assignments across factor levels, by choosing the allocation for the new participant that would lead to the smallest possible degree of imbalance across the set of his or her baseline characteristics [6]. More specifically, in stratified randomization an algorithm is applied to distribute participants to each stratum. Minimization, on the other hand, enables the control of imbalance in covariates not included in the stratification algorithm, such as unknown baseline differences in the outcome measures [24]. In cases of unplanned changes in the study, even re-randomization is recommended before breaking the blind method of the treatment in clinical trials [25].

Balanced treatment groups should be comparable in most relevant aspects except for the specific component applied in the intervention group. However, comparability between the study treatment groups depends on the phenomenon studied. Gender, for example, is important with respect to cardiovascular fitness and to other physical measurements such as static and dynamic balance [13], but is independent of global cognitive functioning. That is, experimental groups do not need to be identical in any respect; it suffices that they are alike with respect to the outcome variable under study [5]. On the other hand, more so than gender, age is quite often considered a moderating variable in clinical trials.

### Assessing the probabilities of inequalities after simple randomization in typical functional tests in small samples of aging populations – practical examples

Based on the R scripts proposed in our previous article [8], we calculated the probabilities of inequalities after simple randomization in selected typical functional tests in small samples of aging populations. The tests included were: Timed Up and Go, Walking Speed, Functional Reach, Sit-to-Stand, Handgrip Strength and Unipedal Balance (one-leg stance). We based our calculations on means and SDs reported in the literature for these tests. Tables 2 and 3 present our calculations for men and women, respectively. It should be noted that in these calculations, we suggested a threshold for accepting the probability of inequality (10% or 15% or 20%), above which we think it may be too risky to use simple randomization. However, such a threshold should be determined by researchers based on the specific research domain, previous experience and previous results.

**Table 2:** The probabilities of inequalities after simple randomization in groups of older **men,** based on scores of selected functional tests

				Probability for inequalities between experimental groups (mean difference ± SD)		
Functional test	Sample	Expected mean ± SD	Participants per group	10% difference	15% difference	20% difference
Timed Up and Go	60+ years	10.8 ± 2.5 s Ibrahim et al., 2017 [26]	15	24.0% (1.5 ± .4)	7.8% (2.0 ± .3)	2.0% (2.5 ± .3)
			20	17.5% (1.5 ± .3)	4.3% (1.9 ± .3)	.7% (2.4 ± .2)
			25	13.0% (1.4 ± .3)	2.3% (1.9 ± .2)	.3% (2.3 ± .2)
Normal Walking Speed	70–79 years	120.0 ± 21.0 cm/s Atkinson, 2007 [27]	15	11.7% (15.3 ± 2.9)	2.0% (20.5 ± 2.4)	.2% (25.9 ± 1.9)
			20	7.1% (14.6 ± 2.4)	.7% (19.9 ± 2)	.03% (24.9 ± 1.4)
			25	4.5% (14.2 ± 2.0)	.3% (19.8 ± 1.8)	.006% (25.1 ± 1.1)
Functional Reach	Hypertensive 80.9 ± 4.3	29.3 ± 7.5 cm Bohannon et al., 2017 [28]	15	28.2% (4.3 ± 1.2)	11.0% (5.5 ± 1.0)	3.3% (6.8 ± .9)
			20	22.0% (4.1 ± 1.0)	6.5% (5.3 ± .9)	1.4% (6.6 ± .7)
			25	16.9% (3.9 ± .8)	3.9% (5.1 ± .7)	.6% (6.4 ± .6)
Sit-to-Stand	60–94 years	14.2 ± 4.6 reps/30s Rikli and Jones, 1999 [29]	15	39.9% (2.4 ± .8)	20.9% (2.9 ± .7)	9.3% (3.5 ± .6)
			20	32.8% (2.2 ± .7)	14.5% (2.8 ± .6)	5.2% (3.4 ± .5)
			25	27.5% (2.1 ± .6)	10.4% (2.7 ± .5)	3.0% (3.3 ± .4)
Handgrip Strength	75–79 years	81.9 ± 9.94 pounds Jansen et al., 2008 [30]	15	2.4% (9.4 ± 1.2)	.07% (13.1 ± .9)	0% (NA)
			20	.9% (9.1 ± .9)	.01% (12.8 ± .6)	0% (NA)
			25	.4% (9.0 ± .8)	.002% (12.26 ± .1)	0% (NA)
Unipedal Balance Test^a^	65–69 years	26.3 ± 18.4 s Lohne-Seiler et al., 2016 [31]	15	64.1% (9.7 ± 4.5)	48.4% (11.1 ± 4.2)	35.1% (12.7 ± 4)
			20	59.3% (8.7 ± 3.8)	42.1% (10.3 ± 3.5)	28.6% (11.8 ± 3.3)
			25	54.6% (8.1 ± 3.4)	36.6% (9.7 ± 3.1)	23.0% (11.2 ± 2.8)

^a^Calculated with the assumption of non-normal distribution due to the large SD

**Table 3:** The probabilities of inequalities after simple randomization in groups of older **women,** based on scores of selected functional tests

				Probability for inequalities between experimental groups (mean difference ± SD)		
Functional test	Sample	Expected mean ± SD	Participants per group	10% difference	15% difference	20% difference
Timed Up and Go	60+ years	11.5 ± 2.7 s Ibrahim et al., 2017 [26]	15	24.3% (1.6 ± .4)	8.2% (2.1 ± .4)	2.0% (2.6 ± .3)
			20	18.2% (1.5 ± .4)	4.4% (2.0 ± .3)	.8% (2.5 ± .3)
			25	13.3% (1.5 ± .3)	2.4% (2.0 ± .3)	.3% (2.5 ± .2)
Normal Walking Speed	70–79 years	111.0 ± 21.0 cm/s Atkinson, 2007 [27]	15	14.9% (14.5 ± 3.1)	3.1% (19.4 ± 2.6)	.4% (24.2 ± 2.3)
			20	9.5% (13.8 ± 2.5)	1.3% (18.7 ± 2.0)	.1% (23.7 ± 1.8)
			25	6.2% (13.4 ± 2.1)	.5% (18.3 ± 1.8)	.01% (22.7 ± 1.5)
Functional Reach	Hypertensive 80.3 ± 3.9 years	26.1 ± 6.5 cm Bohannon et al., 2017 [28]	15	27.4% (3.8 ± 1.0)	10.0% (4.9 ± .9)	2.8% (6.0 ± .8)
			20	20.4% (3.6 ± .9)	5.7% (4.7 ± .7)	1.2% (5.8 ± .6)
			25	15.8% (3.4 ± .7)	3.3% (4.6 ± .6)	.5% (5.7 ± .5)
Sit-to-Stand	60–94 years	12.7 ± 4.0 reps/30s Rikli and Jones, 1999 [29]	15	38.8% (2.1 ± .7)	19.4% (2.6 ± .6)	8.5% (3.1 ± .6)
			20	31.8% (1.9 ± .6)	13.5% (2.4 ± .5)	4.7% (3.0 ± .4)
			25	26.3% (1.8 ± .5)	9.3% (2.4 ± .4)	2.6% (2.9 ± .4)
Handgrip Strength	75–79 years	48.2 ± 10.3 pounds Jansen et al., 2008 [30]	15	20.0% (6.6 ± 1.6)	5.6% (8.6 ± 1.3)	1.1% (10.7 ± 1.1)
			20	13.9% (6.3 ± 1.3)	2.7% (8.3 ± 1.1)	.34% (10.5 ± 1.0)
			25	9.7% (6.0 ± 1.1)	1.3% (8.1 ± .9)	.1% (10.3 ± .8)
Unipedal Balance Test^a^	65–69 years	28.2 ± 18.2 s Lohne-Seiler et al., 2016 [31]	15	64.1% (9.8 ± 4.6)	48.6% (11.3 ± 4.3)	35.3% (12.8 ± 4.0)
			20	59.0% (8.9 ± 3.9)	42.2% (10.4 ± 3.6)	28.1% (12.0 ± 3.4)
			25	55.0% (8.2 ± 3.4)	36.4% (9.8 ± 3.1)	22.9% (11.4 ± 2.9)

^a^Calculated with the assumption of non-normal distribution due to the large SD

For example, in samples of 15 participants per group there is a probability of 24% that there will be a 10% difference between groups in Timed Up and Go in both women and men. In Sit-to-Stand there is a probability of almost 40% (Tables 2 and 3). Special attention should be attributed to balance measurements known to produce large standard deviations, indicating substantial significant variability. These large standard deviations are clearly demonstrated in a wide range of normative data on the unipedal balance test [12]. Therefore, it is not surprising that we calculated a probability of 23% for having a 20% difference between groups of 25 participants in both genders (Tables 2 and 3), and a probability of 64% (!) for having a 10% difference in groups of 15 participants (Tables 2 and 3).

Clearly, it can be argued that research should be conducted with large samples to ensure equal groups. However, in most cases this is quite difficult, especially in intervention studies in older populations. The recruitment is difficult and the rate of attrition is high. Consequently, intervention studies published in journals with a relatively high impact factor are based on small groups. For example, Voelcker-Rehage et al. [32] based their study on two groups of 16 and 17 participants, Rehfeld et al. [21] on 12 and 14, and Eggenberger et al. [33] on 19 and 14. Furthermore, this last study provided support for the relatively small sample by conducting a power analysis. This analysis had revealed a sample size of 17 participants per group based on α-level of 0.05 and effect size of 0.25. It should be noted that a power analysis is quite acceptable in research as a criterion for determining samples size. On the other hand, this criterion does not guarantee equal groups.

A researcher can calculate the number of participants required for creating equal groups for a specific study. In order to illustrate these calculations, we used the same examples provided in Tables 2 and 3, which included the expected means (±SDs) in typical, widely used functional tests for older adults. Table 4 presents our calculations. In the Time-Up-and Go test, for example, in order to allow for no more than 10% difference 44 women and 42 men are recommended in a group, and in the unipedal balance test, 264 women and 262 men in a group, which is clearly unrealistic. If creating equal groups by increasing the sample size is unachievable, it is our recommendation to examine the probability of inequality and possibly acquire an alternative type of randomization.

**Table 4:** Number of participants required to reach equal groups at baseline in simple randomization (values are based on the expected mean ± SD for men and women presented in Tables 2 and 3)

Functional Test	Sample	Allowed difference between groups	# of men required per group^a^	# of women required per group^a^
Timed Up and Go	60+ years	10%	42	44
		15%	19	19
		20%	11	11
Normal Walking Speed	70–79 years	10%	24	28
		15%	11	13
		20%	6	7
Functional Reach	Hypertensive 80.3 ± 3.9 years	10%	51	48
		15%	23	22
		20%	13	13
Sit-to-Stand	60–94 years	10%	82	77
		15%	37	35
		20%	21	20
Handgrip Strength	75–79 years	10%	12	35
		15%	6	16
		20%	3	9
Unipedal Balance Test^b^	65–69 years	10%	262	264
		15%	117	118
		20%	66	67

^a^Probability of no more than 5% inequality

^b^Calculated with the assumption of non-normal distribution due to the large SD

### Summary and recommendations

An experiment with random allocation to groups based on equal chance is often referred to as the gold standard of research. However, an equal chance to be allocated to any group does not guarantee group equality. Imbalanced groups may occur, particularly when there are relatively few participants enrolled in a trial.

In the exercise sciences, age is a moderating variable in almost all areas of research, primarily in relation to physical variables but also to psychological, social and behavioral variables, which are often studied in relation to exercise or fitness. Studies focusing exclusively on older adults have found that variability shows a quantitative increase in advanced age. Given this increased variability in old age, it may be possible that experimental groups of a small sample size (e.g. 15–30 participants per group) will have baseline differences in the outcome measures as well as in relevant covariates. Evidently, a researcher should strive for a sample size large enough to warrant equality. However, this is quite challenging in intervention studies in aging populations. It is therefore recommended to examine the probability that simple randomization will lead to group differences at baseline.

Imbalances between groups can be reduced in studies with a small sample size by restricting the randomization procedure, which means that that simple randomization is applied within defined groups of participants. Covariate-adaptive randomization is the most commonly used procedure for creating balance in relevant covariates across treatment groups, and the two leading techniques of covariate-adaptive randomization are stratified (block) randomization and minimization. R scripts for calculating probabilities of inequalities, and the number of participants required for creating equal groups by simple randomization have been proposed in a previous article [8]. Additional practical information on assessing and implementing randomization techniques has recently been published [34].

And last but not least – in this commentary we discussed the randomization process in interventional studies, elaborating on inter-individual variability typical to aging, which may cause unequal groups at baseline. However, as age increases, both inter- and intra-individual variability are increasing [20]. The intra-individual variability may affect the relationship between pre- and post-tests in interventional studies as well. If participants perform differently in a single task on different occasions, the chances to observe true changes as a result of an intervention is reduced. Strategies to deal with this issue should be discussed in future research.

## Conclusion

Given this increased variability in old age – it is recommended in small groups of older adults to examine the probability of baseline differences before conducting a simple randomization and, if necessary, to apply a restricted randomization technique.

## Data Availability

Not Applicable
